# Rehabilitation and Return to Sport of Female Athletes

**DOI:** 10.1016/j.asmr.2021.09.040

**Published:** 2022-01-28

**Authors:** Arianna L. Gianakos, Adam Abdelmoneim, Gino Kerkhoffs, Mary K. Mulcahey

**Affiliations:** a Department of Orthopaedic Surgery, Harvard-Massachusetts General Hospital, Boston, Massachusetts, U.S.A.; b Department of Orthopaedic Surgery, Amsterdam Movement Sciences, Amsterdam University Medical Center, Academic Medical Center, University of Amsterdam, Amsterdam, The Netherlands; c Royal College of Surgeons in Ireland, Dublin, Ireland; d Department of Orthopaedic Surgery, Tulane University School of Medicine, New Orleans, Louisiana, U.S.A.

## Abstract

The increase in female participation in athletics over the past decade has been accompanied by an increase in injury rates as a result of higher demands placed on athletes. Although previous studies have shown that anatomic, biomechanical, hormonal, and psychological factors may play a role in differences between men and women that can influence injury risk in athletes, there is still a lack of understanding of sex-related mechanisms of injury, guidelines, and prevention strategies. This article provides an overview of common injuries affecting female athletes. We present guidelines for upper- and lower-extremity injury rehabilitation, focusing on considerations specific to the female athlete with the goal to facilitate a safe return to sports.

**Level of Evidence:**

Level V, expert opinion.

Female participation in athletics has increased over the past decade, with a rapid rise in participation in sports previously deemed “male dominated.”^1^ The higher demands placed on athletes have been accompanied by increased injury rates, which may be the result of a lack of understanding of sex-related mechanisms of injury, guidelines, and prevention strategies. Previous studies have shown that anatomic, biomechanical, hormonal, and psychological factors may play a role in differences between men and women that can influence injury risk in athletes.^1^ Therefore, a better understanding of these factors is needed to more appropriately manage and prevent sports-related injuries in the female athlete. In addition, rehabilitation strategies should be individually tailored to return the athlete back to her sport safely and efficiently without a repeated injury. A multidisciplinary approach may be beneficial to appropriately address nutritional, metabolic, endocrine, and psychological concerns. Therefore, sports physicians should collaborate with coaches, athletic trainers, nutritionists, and mental health professionals. This article will provide an overview of common injuries affecting female athletes. We describe various modalities and rehabilitation strategies to facilitate a safe return to sport for female athletes.

## Common Injuries Affecting Female Athletes

Increased sports participation has led to an increased rate of injury in female athletes, and previous studies have shown an increased risk of certain injuries in women when compared with men.^1^^,^^2^ For example, overuse injuries are more common in female athletes than in similarly aged male athletes.^2^ Common injuries affecting female athletes include anterior cruciate ligament (ACL) rupture, patellofemoral pathology, ankle sprain and instability, and shoulder instability (Table 1).

**Table 1:** Common Injuries Affecting Female Athletes

Overuse injury
Anterior cruciate ligament rupture
Patellofemoral injury
Ankle sprain
Chronic ankle instability
Shoulder instability

ACL tears are 2 to 8 times more common in female athletes than in their male counterparts.^3^ Patellofemoral pathology is also more common in female athletes than in male athletes, with patellofemoral syndrome being 2.23 times more likely to develop in female athletes.^4^ Previous studies have reported that female athletes often require surgical intervention more frequently than male athletes for patellar instability.^5^ Anatomic and hormonal differences, biomechanical factors, neuromuscular factors, genetic predisposition, and even psychological assessment of competitive ability have all been suggested to play a role in the developmental differences in injuries between male and female athletes.^6^^,^^7^ Female athletes tend to have an increased quadriceps angle, dynamic knee valgus, quadriceps dominance, and hamstring weakness when compared with men.^2^ Understanding these differences is important to improve knee injury prevention protocols when training and rehabilitating female athletes.

Previous studies have shown sex differences in the epidemiology of ankle injuries, particularly ligament sprains and instability.^8^^,^^9^ Swenson et al.^8^ reported that female athletes had a significantly higher ankle sprain injury rate compared with male athletes in comparable sports including soccer, volleyball, basketball, baseball or softball, lacrosse, swimming and diving, and track and field. Lateral ankle ligament injuries occur more often in women, whereas studies have shown that men have higher rates of medial ankle sprains.^9^ Caldemeyer et al.^10^ conducted a systematic review examining the sex-specific effectiveness of neuromuscular training (NMT). They found that evidence supports the efficacy of NMT in preventing ankle sprains in female athletes and that investigators should consider using comprehensive approaches that incorporate strength, balance, plyometric, and agility training given that the studies with significant findings created comprehensive NMT programs. Therefore, proper evaluation of ankle injuries and individualized sports-specific rehabilitation protocols are useful for improving both neuromuscular control and proprioception when athletes return to sport.

Although traumatic shoulder instability is more prevalent in male athletes, female athletes often present with multidirectional instability and studies have shown that female athletes have poorer outcomes after surgical management of shoulder instability.^11^^,^^12^ Increased ligamentous laxity exhibited by female patients can be challenging for surgeons because these patients may require additional tightening procedures including rotator interval closure and capsular shift, therefore possibly leading to poorer outcomes.^2^ Raynor et al.^13^ showed that female patients had a 40% risk of postoperative instability compared with a 22% risk of postoperative instability in male patients. Physicians and physical therapists should keep this in mind, particularly when developing a rehabilitation protocol for sports that require repetitive overhead-throwing motions such as baseball, volleyball, football, and javelin throwing.^2^

## Therapeutic Modalities

Several innovative therapeutic modalities have been developed and used to reduce rates of injury and minimize the amount of rehabilitation required for athletes to return to play (RTP) after an injury. One such modality is cryotherapy—a well-established and widespread therapy used in athletes in an attempt to treat soft-tissue injuries and reduce post-training inflammation and pain.^14^^,^^15^ Studies have shown that cryotherapy has an overall positive effect on injured athletes returning to play.^16^ Cryotherapy includes the use of topical cooling methods such as ice packs, ice towels, ice massages, and gel packs, as well as whole-body cryotherapy (WBC) methods including cold water–ice immersion (CWI) and newer commercial WBC devices that work by using cold air currents. A recent systematic review by Jinnah et al.^17^ reported statistically significant findings in terms of decreased subjective muscle soreness and pain levels in athletes who used CWI in comparison to passive recovery. However, it was discovered that longer CWI durations (>10 minutes) were associated with detrimental effects on muscle power and activity. It is also important to note that the current literature has shown that in athletes who use WBC devices, there are limited or mixed results on functional recovery, defined as the return to baseline after exertion of any form in terms of strength, pain, and subjective fatigue.^18^

Another often-performed therapeutic technique is iontophoresis, whereby a direct current of low amperage is applied to the skin by means of bipolar electrodes to increase the permeability of skin and enhance the delivery of transdermal drugs or prodrugs into the deeper tissues such as the muscles or tendons or the synovial fluid.^19^ Iontophoresis allows for a noninvasive alternative to enteral and parenteral administration as a drug-delivery method to pathologic areas with reduced systemic side effects.^19^^,^^20^ Previous systematic reviews and meta-analyses have shown iontophoresis to be more effective than placebo in the treatment of medial and lateral epicondylitis, tendinopathy (e.g., tendinitis), calcifying disorders, knee osteoarthritis, and pain.^19^^,^^20^ There were, however, no statistically significant improvements in patients with carpal tunnel syndrome, plantar fasciitis, or rheumatoid knee disease.^19^

Blood-flow restriction (BFR) therapy is a more recently developed technique often used in the athletic population. BFR involves localized restriction of venous blood outflow from a specific portion of the body using an occlusive pressure device such as a tourniquet or inflatable cuff placed at the proximal end of an extremity. The restriction of venous outflow results in an anaerobic environment, which promotes muscle hypertrophy through cellular signaling and hormonal changes similar to those seen during higher-intensity training with more resistance.^21^ The mechanism by which BFR elicits such cellular and hormonal responses is still unclear. A recent systematic review by Wortman et al.^21^ reported statistically significant increases in strength and sports performance but showed variable results in terms of muscle hypertrophy with the use of BFR training.

Finally, there are several injectable therapies used in the athletic population including platelet-rich plasma therapy, autologous conditioned plasma therapy, mesenchymal stem cell exosome therapy, mesenchymal stem cell–Wharton jelly therapy, gold-induced cytokine therapy, and prolotherapy. These and several other recently developed therapeutic techniques have been seen more prevalently in the recent sports medicine literature and have been gaining popularity with athletes, coaches, physiotherapists, and physicians alike.^22^, ^23^, ^24^, ^25^ These techniques have been used to treat several different pathologies ranging from tendinitis and bursitis to rotator cuff injuries and even chronic back and joint pain, with promising results. Much more research is needed to adequately examine the efficacy of these newer therapies; however, their use is rapidly growing.

## Overview of Rehabilitation

Injury rehabilitation typically involves several sequential phases (Table 2). The initial phases focus on restoring preinjury joint range of motion (ROM) and regaining mobility and flexibility, followed by redeveloping preinjury strength and gradual RTP with activity modification. It is important to note, however, that even though these phases are sequential, they should also be performed simultaneously throughout the rehabilitation process.^26^ Rehabilitation phases are not forgone once an athlete progresses to the next phase; hence, it is essential to maintain a joint’s flexibility via consistent mobility work and stretching while performing strength training after RTP to minimize rates of injury.^26^

**Table 2:** Phases of Rehabilitation

Phase	Description
1	Restore preinjury joint ROM; focus on regaining mobility and flexibility.
2	Restore preinjury strength
3	Perform sport-specific training

ROM, range of motion.

Once pain-free ROM and mobility have returned to or are near baseline, the athlete can advance to the strength redevelopment phase of rehabilitation. An athlete’s baseline strength level and sport are integral factors to consider when developing a strength training program. A strength training program should maximize the use of compound movements if the athlete’s injuries allow for it. Compound movements facilitate functional strength development by having the athlete perform complex movements that involve multiple joints and muscle groups (e.g., squat, dead lift, bench press, and clean and jerk). These movements are more neurally demanding and allow for larger loads to be lifted, leading to greater neuromuscular development. Compound training has also been proved more effective for increasing overall muscular strength, promoting hypertrophy, and reducing rates of injury and reinjury in athletes when compared with isolation movements.^27^ These compound movements also translate to sport much more effectively than the performance of isolation training alone.^27^

Once these initial phases have been completed, sport-specific exercise selection then allows for adaptations tailored to the athlete’s sport. Given that the risk factors for sustaining an injury vary based on sport, tailoring the rehabilitation program to the athlete’s sport can result in better outcomes.^27^^,^^28^

## Upper-Extremity Rehabilitation

For the upper extremity (UE), there are several devices that can allow for self-myofascial release. The Graston technique, massage balls, and massage guns are the primary methods by which UE self-myofascial release is performed because foam rollers are generally larger and more suited for the lower extremity (LE) and posterior chain.^27^^,^^28^ Static and dynamic stretching are both useful in restoring preinjury ROM. Stretching exercises should be performed with progressively increasing intensity and duration to establish soft-tissue extensibility.

Once ROM is re-established, the strength training phase of rehabilitation should start with low-intensity exercises. Single arm–throwing athletes typically have significant asymmetry in shoulder strength and flexibility, which can frequently cause injury.^28^ Given that the dynamic stability of the shoulder is highly dependent on the rotator cuff and surrounding musculature, a significant portion of the shoulder rehabilitation program should be focused on developing these muscle groups.

Although there are some compound exercises that allow for shoulder development (e.g., overhead press and bench press), these can be challenging to perform, especially by female athletes with pre-existing shoulder injuries. Instead, shoulder strength can be developed by performing isolation exercises with lighter loads such as light dumbbells, resistance bands, or cable machines. Such exercises include lateral raises, internal and external shoulder rotation, and the Y-T-W-L exercise (a popular shoulder exercise performed while prone with the UE taking the shape of each letter).^28^ The posterior deltoid, trapezius, and latissimus dorsi, though often overlooked in shoulder training, are also important for stabilization. These can be targeted using cable machine face pulls, shrugs, and pull downs, respectively. Shoulder rehabilitation programs should have an equal balance between the stability and the mobility and/or flexibility of the joint, given that overdevelopment of these muscles can be detrimental to the ROM of the shoulder. Hence, in patients with shoulder injuries, mobility training and strength training are often performed simultaneously.^28^

Elbow and wrist training plays an important role, particularly in participants in throwing sports, in reducing the risk of medial and lateral epicondylitis. Resistance training has proved to be an effective method in strengthening the muscles of the forearm and reducing the incidence of epicondylitis.^27^ Distal-arm endurance development is also vital in participants in sports that require holding heavier loads for prolonged periods, such as lacrosse and hurling.^28^, ^29^, ^30^

Finally, grip training is widely recognized as a crucial part of UE development and rehabilitation. Many recently developed creative techniques target this specifically, including the use of handgrip tools, kettle bells, farmer’s walks, and TheraBand Flexbars. Grip training can also be incorporated into general strength training by using handles that are more difficult to grasp, forcing the development of strength and dexterity in the muscles responsible for grip. This can be further extended into the specific development of the intrinsic muscles of the hand by using finger exercise methods, such as finger extension resistance bands and finger exercisers.^29^, ^30^, ^31^

UE orthotics and taping can be a very useful tool during the RTP phase because they can provide an extrinsic support that reduces the risk of reinjury. Splints and braces are particularly useful in the treatment of elbow, wrist, and finger injuries because they can effectively limit ROM and protect against hyperextension and valgus-varus stresses. Kinesio Taping has been shown to be effective in improving shoulder proprioception and reducing the risk of shoulder injury.^26^^,^^32^

## Lower-Extremity Rehabilitation

LE rehabilitation follows many of the same principles as UE rehabilitation. The added difficulty, however, is that effective strength training in the LE, unlike in the UE, is heavily dependent on compound movements (e.g., squat and dead lift), which often involve the UE and the core. This can be challenging because injuries involving other joints in the UE or core might limit an athlete’s ability to adequately perform LE strength rehabilitation.

To some degree, this can be overcome by performing isolation resistance training that focuses on monoarticular movements, but unfortunately, this is not a very effective method of building LE strength or functional rehabilitation when used as a monotherapy. In addition, addressing lower-limb coordination and proprioception can be challenging during rehabilitation. However, it is of utmost importance to avoid reinjury, particularly in female athletes with knee and ankle injuries (e.g., ACL rupture), which cannot be addressed with isolation training.^27^^,^^33^^,^^34^

Dynamic stabilization of the knee is primarily achieved via the hamstrings and quadriceps. The functional hamstring-to-quadriceps (H/Q) ratio—the functional ratio of eccentric hamstring to concentric quadriceps moments (extension) and concentric hamstring to eccentric quadriceps moments (flexion)—has been described as one of the integral principles that determine the degree of strain on the ACL.^33^^,^^35^ Studies have shown that H/Q muscular imbalances increase ACL strain and the risk of injury, as well as reinjury.^33^^,^^35^^,^^36^ This is particularly important for injured female athletes who tend to have weaker hamstrings when compared with male athletes, which is one of the factors increasing the risk of ACL injury and reinjury in this specific population.^36^

At high knee flexion angles, a low H/Q ratio may represent a compromised ability of the hamstrings to stabilize the knee joint throughout the full ROM. Near full knee extension, shifts in favor of the knee flexors may represent an attempt to stabilize the knee at the angle of greatest ACL strain.^35^ Dedinsky et al.^37^ reported that exercises that facilitate functional and optimal hamstring and quadriceps co-activation such as double-leg squats, single-leg squats, lateral step-up exercises, and other squat variations help decrease the risk of ACL injury in healthy female individuals. Biomechanical data from patients undergoing ACL reconstruction also suggest that hamstring co-contraction with the quadriceps is effective in reducing excessive forces on the ACL particularly between 15° and 60° of knee flexion.^38^ Finally, female athletes who participate in high-risk sports such as basketball and soccer have a 2- to 8-fold higher rate of ACL injury than male athletes.^39^ There have been several kinematic and biomechanical laboratory studies that have shown significant sex-specific differences between male and female individuals. These differences might explain why women are at more risk of noncontact injuries during sports and emphasize the need for muscle development in female athletes.^39^^,^^40^ Kinematic studies have shown that female athletes can injure the ACL during simple deceleration maneuvers whereas male athletes typically require more strenuous mechanisms of injury such as jumping.^39^ Female athletes frequently land with higher knee abduction moments, which is the predominant risk factor for ACL injury in female individuals.^39^ Some of the factors that contribute to this include increased joint laxity, knee recurvatum or hyperextension, increased tibial slope, and changes in estrogen levels.^39^

Given the crucial protective value of the hamstrings and quadriceps in reducing the risk of ACL injuries, developing functional strength is at the core of LE rehabilitation. A highly cited study by Renström et al.^41^ published in 1986 was one of the first studies to report not only that hamstring strength training is safe in ACL reconstruction rehabilitation but that isometric hamstring activity decreased ACL strain relative to the passive normal strain at all positions tested. These results have been replicated in many studies since.^42^^,^^43^ The hamstring and quadriceps thus act as the primary dynamic stabilizers of the knee in the RTP phase of rehabilitation because the use of braces in patients with ACL injuries has not proved significantly beneficial.^33^

Similarly, rehabilitation after an acute ankle sprain or chronic ankle instability includes early functional rehabilitation, which focuses on increasing ROM, resistance strength training, proprioception exercises, and sport-specific training.^44^ When an athlete is deemed suitable to RTP after an ankle injury, the RTP should occur gradually with the added protection of bracing designed to limit or improve ROM and to provide mechanical stability.^44^

## Considerations Specific to Female Athletes

Anatomic differences, hormonal differences, biomechanical factors, neuromuscular factors, genetic predisposition, and even psychological assessment of competitive ability have all been suggested to play roles in the differences between male and female athletes.^6^^,^^7^ Structural differences between men and women have been well documented, with women exhibiting greater static external knee rotation alignment, greater active hip internal rotation, and increased hip and pelvic widths, which are thought to contribute to increased genu valgum.^45^^,^^46^ In addition, women tend to have an increased quadriceps angle, dynamic knee valgus, quadriceps dominance, and hamstring weakness when compared with men.^2^ Previous studies have also shown that men and women exhibit mechanical differences during running, cutting, and landing.^6^ Finally, hormonal and neuromuscular differences between men and women may play a role in the increased ligamentous laxity experienced by women when compared with men. These structural differences combined with differences in muscle recruitment and timing of muscle activation can lead to an increased risk of the injuries previously discussed and, therefore, should all be taken into consideration when developing a protocol for rehabilitation and return to sport.

Although there is a paucity of literature evaluating outcomes of various gender- and sex-specific rehabilitation strategies, many protocols address the 4 following deficits typically seen in the injured female athlete: (1) ligament dominance, (2) quadriceps dominance, (3) leg dominance, and (4) trunk dominance.^6^ Core strengthening is recommended after injury and includes exercises to strengthen the abdominal muscles, hip muscles, and lumbar spine muscles. Jeong et al.^47^ performed a biomechanical analysis evaluating the influence of core stability on LE injuries and found that core strength training altered the motor control strategies and joint kinematics for the trunk and the LE by increasing the trunk flexion angle, vastus medialis–to–vastus lateralis activation ratio, and H/Q activation ratio and reducing the knee valgus and hip adduction angles. Plyometric exercises should also be incorporated into the training regimen, focusing on jumping, landing, and cutting biomechanics, to decrease forces on the knee and normalize lower-limb alignment.^6^ The goal of these exercises is not only to help return athletes to their sport but also to prevent future injuries, particularly knee injuries. Finally, it is well known that women have a higher propensity to experience an ankle or knee ligament injury when compared with men.^2^^,^^6^ Balance and proprioception training can assist in normalizing afferent input and improve neuromuscular responses during athletic activities, further reducing injury risk and improving the rate of return to sport after injury.

## Psychological Considerations in Return to Sport in Female Athletes

Psychological factors including self-efficacy, identity, and fear of reinjury may all play a role in an athlete’s ability to return to sport.^48^ Previous studies have reported that female athletes recovering from injury are often self-directed and exhibit greater levels of anxiety concerning an injury’s impact on their lives and a loss of physical self-worth with injury when compared with their male counterparts.^49^, ^50^, ^51^ Gennarelli et al.^52^ conducted a systematic review evaluating outcomes after psychosocial interventions to help facilitate recovery after musculoskeletal sports injuries and showed positive mood changes, improved pain management, and better exercise compliance and rehabilitation adherence when the following interventions were incorporated during rehabilitation: relaxation and/or guided imagery, positive self-talk, goal setting, counseling, emotional and/or written disclosure, and modeling videos. Therefore, addressing the psychological concerns after injury during the period of rehabilitation is important to better facilitate return to sport in female athletes.

## Conclusions

Increased female sports participation has been accompanied by an increase in injury rates. Understanding how female athletes differ from their male counterparts regarding the mechanism of injury and recovery is important to tailor rehabilitation strategies to the individual female athlete. This article shows the importance of rehabilitation considerations specific to female athletes to more effectively return them to their sports efficiently and safely.

## Supplementary Data

ICMJE author disclosure forms
